# Exploring the causal pathway from ischemic stroke to atrial fibrillation: a network Mendelian randomization study

**DOI:** 10.1186/s10020-019-0133-y

**Published:** 2020-01-15

**Authors:** Lei Hou, Mingqing Xu, Yuanyuan Yu, Xiaoru Sun, Xinhui Liu, Lu Liu, Yunxia Li, Tonghui Yuan, Wenchao Li, Hongkai Li, Fuzhong Xue

**Affiliations:** 1 Healthcare Big Data Institute of Shandong University, Jinan, 250000 People’s Republic of China; 2 Department of Epidemiology and Health Statistics, School of Public Health, Shandong University, 44 Wenhua West Road, Jinan, 250000 Shandong province People’s Republic of China; 3 Bio-X Institutes, Key Laboratory for the Genetics of Developmental and Neuropsychiatric Disorders (Ministry of Education), Shanghai Jiao Tong University, Shanghai, 200030 People’s Republic of China; 4 School of Mathematical Sciences, Peking University, Beijing, People’s Republic of China 100000

**Keywords:** Ischemic stroke, Atrial fibrillation, Network Mendelian randomization, Bidirectional causality

## Abstract

**Background and purpose:**

Previous studies have found ischemic stroke is associated with atrial fibrillation. However, the causal association between ischemic stroke and atrial fibrillation is not clear. Furthermore, the network relationship among ischemic stroke, atrial fibrillation and its risk factors need further attention. This study aims to examine the potential causal association between ischemic stroke and atrial fibrillation and further to explore potential mediators in the causal pathway from ischemic stroke to atrial fibrillation.

**Methods:**

Summary statistics from the ISGC (case = 10,307 and control = 19,326) were used as ischemic stroke genetic instruments, AFGen Consortium data (case = 65,446 and control = 522,744) were used for atrial fibrillation, and other consortia data were used for potential mediators (fasting insulin, white blood cell count, procalcitonin, systolic and diastolic blood pressure, body mass index, waist circumference, and height). Under the framework of network Mendelian randomization, two-sample Mendelian randomization study was performed using summary statistics from several genome-wide association studies. Inverse-variance weighted method was performed to estimate causal effect.

**Results:**

Blood pressure mediates the causal pathways from ischemic stroke to atrial fibrillation. The total odds ratio of ischemic stroke on atrial fibrillation was 1.05 (95% confidence interval [CI], 1.02 to 1.07; *P* = 1.3 × 10^−5^). One-unit increase of genetically determined ischemic stroke was associated with 0.02 (DBP: 95% CI, 0.001 to 0.034, *P* = 0.029; SBP: 95% CI, 0.006 to 0.034, *P* = 0.003) upper systolic and diastolic blood pressure levels. Higher genetically determined systolic and diastolic blood pressure levels were associated with higher atrial fibrillation risk (DBP: RR, 1.18; 95% CI, 1.03 to 1.35; *P* = 0.012. SBP: RR, 1.18; 95% CI, 1.01 to 1.38; *P* = 0.04). Specially, we also found the bidirectional causality between blood pressure and ischemic stroke.

**Conclusions:**

Our study provided a strong evidence that raised blood pressure in stroke patients increases the risk of atrial fibrillation and active acute blood pressure lowering can improve the outcome in ischemic stroke patients.

## Introduction

Atrial fibrillation (AF) is the most common sustained cardiac arrhythmia encountered in clinical practice and is associated with increased risk of stroke, dementia, falls, and death, among other outcomes (Chugh et al. 2014; Cerasuolo et al. 2017a). AF is a common cause of stroke, which is biologically plausible. However, AF is sometimes detected after ischemic stroke (IS), and one in every four cases of AF begins with continuous ECG monitoring shortly after the onset of symptoms (Cerasuolo et al, 2017b). The current guidelines recommend anticoagulant therapy for every patient with recurrent AF after stroke. However, in some cases, AF detected after acute IS may be shortlasting and perhaps a nonrecurrent autonomic and inflammatory epiphenomena of stroke, so some patients may face an unnecessary risk of bleeding (Haeusler et al, 2018). In addition, more knowledge is needed to determine the mediating mechanism of AF detected after IS, and to effectively prevent the occurrence of new-onset AF after IS (Sposato et al. 2014; Scheitz et al. 2015; Fauchier et al. 2017). Previous population-based observational studies found that IS has been established as a risk factor of AF (Rizos et al. 2015; Luo et al, 2018). However, the causal association between IS and AF has not been studied yet. Furthermore, the potential pathways involved in the association from IS to AF remain unclear. In the past few years, several traditional and newly emerging risk factors for AF, including thyroid function (Roberts 2019), glycemic traits (Kokubo et al. 2015; Chen et al. 2019), inflammation (Paquet et al. 2018; Karam et al. 2017) and obesity (Tikkanen et al, 2019; Aune et al. 2017; Frost et al. 2014; Karas et al. 2016; Nattel 2017) are also closely related to IS. Thus, these may act as potential mediators that lie in the pathway from IS to increased risk of AF.

In this study, we evaluated the potential causal roles of IS in AF and explored the potential mediators involved in the causal association between IS and AF in a network Mendelian randomization analysis framework based on the summarized genome-wide association study data. The potential mediators include fasting insulin, white blood cell count, procalcitonin, systolic and diastolic blood pressure, body mass index, waist circumference, and height.

## Materials and methods

### Summary of GWAS data

The GWAS summary statistics datasets we used in this study were from the ISGC Consortium (https://strokegenetics.org/) for ischemic stroke (Malik et al, 2016); MAGIC Consortium (http://www.magicinvestigators.org/) for FastingInsulin; GIANT Consortium (https://www.broadinstitute.org/collaboration/giant/index) for body mass index, waist circumference and height (Locke et al, 2015; Shungin et al, 2015; Wood et al, 2014); Neale Lab (http://www.nealelab.is/) for systolic and diastolic blood pressure (Churchhouse & Neale, 2017); Astle W for white blood cell count (Astle et al, 2016); MRC-IEU Consortium (http://www.bristol.ac.uk/integrative-epidemiology/) for procalcitonin (Mitchell et al, 2017); AFGen Consortium (https://www.afgen.org/) for atrial fibrillation (Roselli et al, 2018). They were commonly used in MR analyses and to obtain the associations of genetic variants on IS, risk factors and AF, respectively. Beta coefficients (logOR) and standard errors were obtained for the per allele association of each SNP with all exposures and outcomes from these data sources. The basic characters of these data is briefly presented in Table 1 and Additional file 2: Tables S1-S7 for details. There is no sample overlap between ISGC Consortium and AFGen Consortium. To minimize the bias caused by population stratification, populations of the GWAS summary statistics data were mainly from European ancestry, and some of the GWAS summary statistics were adjusted for the population stratification by principal component analysis (PCA) (Price et al. 2006).

**Table 1:** Summary statistics data sources

Trait	Consortium	Data sources	Total no. or case/control	Ancestry
IS	ISGC	Malik; *Neurology*, 2016	10,307/19326	Caucasians
DBP	UKBiobank	Neale; *Neale Lab*, 2017	317,756	Europeans
SBP	UKBiobank	Neale; *Neale Lab*, 2017	317,754	Europeans
FastingInsulin	MAGIC	Horikoshi; *PLOS Genet*, 2015	24,245	Europeans
BMI	GIANT	Locke; *Nature*, 2015	322,154	Europeans
WC	GIANT	Shungin D; *Natrue*, 2015	224,459	Europeans
Height	GIANT	Wood; *Nature*, 2014	253,288	Europeans
WBC	UK Biobank + INTERVAL + UK BiLEVE	Astle W; *Cell*, 2016	172,435	Europeans
PCT	MRC-IEU	Ben Elsworth; *MRC-IEU*, 2018	3701/459309	Europeans
AF	AFGen	Carolina; *Nature*, 2018	65,446/522744	99.2% European, 0.8% African American

### SNP selection

We used 103 IS-associated single-nucleotide polymorphisms (SNP) (explaining 6.1% of its variance) identified by Malik et al. as genetic instruments. This was conducted by first extracting the effect sizes for SNP associated (*P* ≤ 0.0005) with IS from the summary statistics for AF and its risk factors. As the extracted SNPs for IS might be correlated with each other, we pruned the variants by linkage disequilibrium (LD) (*r*^*2*^ = 0.001, clumping window = 500 kbp). We then organized these SNPs by quantifying the heterogeneity and the proportion of variance explained by the genetic instruments (*R*^2^, estimated from the summary statistics using R package *gtx* in R Version 3.5.3). The method assumes that all valid instrumental variables (IV) should yield the same causal estimate. The associations of each SNP with the outcome should be proportional to their association with IS. Presence of any substantial heterogeneity would be suggestive evidence of pleiotropic effects of the SNPs.

The proportion of variance (*R*^2^) of the trait explained by the genetic instruments will rise with the addition of more SNPs. However, the improvement beyond the optimum number of SNPs in the instrument will come increasingly as a result of heterogeneity. In order to determine the final tally of SNPs for inclusion in genetic instruments for each trait, the process is described as follows (White et al. 2016):

- **Step 1** Choose the SNP that explains the largest proportion of variance (*R*^2^) of the trait as the initial element of the IV set.
- **Step 2** Add a SNP from the remaining SNPs to IV set so that the IV set explains the largest proportion of variance (*R*^2^) of the trait and without heterogeneity.
- **Step 3** Repeat the step 2 until there is heterogeneity.

After the above three steps, the homogeneity assumption is ensured to be satisfied, which means instrumental variables-exposure, instrumental variables-outcome and exposure-outcome relationships with no effect heterogeneity. To some extent, it reduces the occurrence of pleiotropic. In terms of the rule of thumb proposed by Staiger and Stock (1997), *F* statistics of the 103 SNPs we selected are all greater than 10, which means the first core assumption of MR is satisfied and avoids the bias caused by weak instruments (Burgess & Thompson, 2011). Finally, we matched SNPs across the data sources by aligning them to the same effect allele, which were checked for concordance. The details are presented in Additional file 1 and Additional file 2: Tables S8-S11.

### Mendelian randomization

Mendelian randomization (MR) can be used to assess the causal effect of an exposure on an outcome using genetic variants as IVs (Davey Smith &Hemani, 2014; Didelez & Sheehan, 2007; Zheng et al, 2017). Three assumptions of Mendelian Randomization should be satisfied: 1) the genetic variants are associated with the exposure; 2) the genetic variants are not associated with any confounders of the exposure and the outcome; 3) the genetic variants are conditionally independent of the outcome given the exposure and confounders (Burgess et al. 2015b). To perform a two-sample MR analyses using summary statistics (Burgess et al, 2015a), we constructed IVs using multiple genetic variants with an inverse variance weighted method to estimate the causal effect sizes (White et al. 2016).

**Fig. 1:**
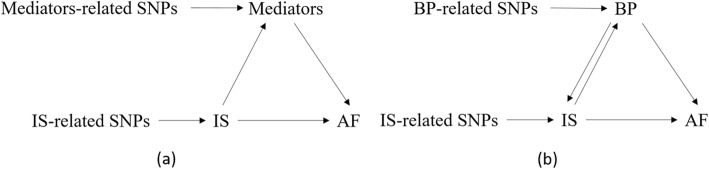
**a** Network Mendelian randomization analysis framework; **b** Graphical diagram of network relationship among IS, AF and BP

We performed network MR to explore the causal pathway from IS to AF. The framework of the network MR analysis (Zhan et al, 2017) is described in Fig. 1a. It consists of three different MR tests that are all described below (I–III) (Burgess et al. 2015b).

I. The causal effect of genetically determined IS on AF is estimated.
II. The causal effects of genetically determined IS on the risk factors for AF are estimated.
III. The causal effects of the possible mediators on AF are estimated.

If causal associations are evaluated in all three steps, the conclusion can be drawn that the specific risk factor is a mediator.

For the first step (I), 103 SNPs of genome-wide significance with IS in the GWAS from the International Stroke Genetics Consortium (ISGC) were used to estimate the causal effect of genetically determined IS on AF using the summary statistics from the AFGen consortium. The second step (II) used the same IVs for IS as described in the first step and estimated the causal effect of genetically determined IS on fasting insulin, body mass index, waist circumference, height, systolic and diastolic blood pressure from the respective GWAS summary statistics. Finally, the third MR analyses (III) were performed for risk factors on AF if IS was shown to have a causal effect on the risk factors in the second test (II). The association between IS and AF was mediated by DBP and SBP were tested in an additional analysis after DBP and SBP were identified as potential mediators. The detailed description of Mendelian Randomization analysis can be referred to the Additional file 1.

### Sensitive analysis

Sensitivity analyses were performed to test the sensitivity of the disproportionate effects of variants and the pleiotropy in MR analysis. These issues were analyzed by leave-one-out validation and MR-Egger regression (Bowden et al. 2015), respectively.

To test the sensitivity of variants, we designed a leave-one-out validation measure. Each SNP was removed from the 103 SNPs to carry out IVW point estimate and then evaluated the influence of each SNP on the results. The fluctuation of the results before and after removing each SNP reflects the sensitivity of this SNP.

For MR-Egger method, we performed weighted linear regression with the intercept unconstrained. The intercept represents the average pleiotropic effect across the genetic variants (the average direct effect of a variant with the outcome). If the intercept differs from zero (the MR-Egger test), then there is the evidence of directional pleiotropy. The Instrument Strength Independent of Direct Effect (InSIDE) assumption needs to be satisfied, which means the effect of genetic variants on the exposure is independent of the direct effects of the genetic variants on the outcome.

Additionally, bidirectional MR (Chung et al. 2001) analyses were performed to examine whether the mediators (systolic and diastolic blood pressure) could causally affect IS by exchanging IS and mediator and using the mediator-associated SNPs as the IV. All statistical analyses were performed using R (version 3.5.3) and R package *TwoSampleMR*. Statistical power was calculated in http://cnsgenomics.com/shiny/mRnd/.

## Results

### Causal association between genetically determined IS and AF

The causal estimate using 103 SNPs as IVs showed that IS will increase the risk of developing AF (odds ratio [OR], 1.12; 95% confidence interval [CI], 1.01--1.22; *P* = 0.019). The intercept of MR-Egger regression for these 103 SNPs was not statistically significant (*P* = 0.397) so there is no evidence of directional pleiotropy. However, the results of leave-one-out indicated the SNP rs12646447 had strong influence on the estimation of causal association (Additional file 1: Figure S6). After removing rs12646447, we found that IS increased the risk of developing AF (odds ratio [OR], 1.05; 95% confidence interval [CI], 1.02--1.07; *P* = 1.3 × 10^−5^). Details are presented in Table 3 and Additional file 1: Figure S4.

### Causal association between genetically determined IS and risk factors

The causal estimates between genetically determined IS and risk factors, including fasting insulin, body mass index, waist circumference, height, systolic and diastolic blood pressure are listed in Table 2. The MR analyses showed that IS was associated with body mass index, systolic and diastolic blood pressure. The intercept of MR-Egger regression for these 102 SNPs in each trait was not statistically significant except BMI. Because of pleiotropic effects of 8 SNPs (rs17696736, rs3184504, rs11250077, rs2074314, rs7123414, rs10172342, rs1483968, rs1483968), they were then excluded from the analysis for BMI. The MR-Egger test for testing the remaining SNPs was not statistically significant (*P* = 0.08). Analysis of the remaining SNPs yielded an effect size of IS on BMI was − 0.00248 (95% CI, − 0.013358 to 0.008398; *P* = 0.654). As a result, the MR analyses showed that IS had causal association with systolic and diastolic blood pressure. Details are presented in Table 2 and Additional file 1: Figure S5.

**Table 2:** Causal estimates for the association between IS and risk factors for AF

Trait	Causal estimate		
	Beta	SE	PValue
BMI	−0.00248	0.00555	0.654
height	−0.00767	0.00706	0.276
WC	−0.00612	0.00660	0.354
FastingInsulin	0.00104	0.00560	0.851
DBP	0.01866	0.00853	0.029
SBP	0.02094	0.00708	0.003
WBC	0.02446	0.01300	0.060
PCT	0.00035	0.00026	0.177

### Causal association between genetically determined systolic and diastolic blood pressure and AF

Based on the first two steps of Network Mendelian randomization analysis, systolic and diastolic blood pressure were suspected to be potential mediators from higher DBP and SBP to increased risk of AF. Further, we evaluated whether DBP and SBP were associated with AF using MR analysis. We used 131 SNPs and 105 SNPs associated with DBP and SBP as the IVs, respectively. Higher DBP and SBP levels were associated with increased risk of AF (DBP: OR, 1.18; 95% CI, 1.03--1.35; *P* = 0.012. SBP: OR, 1.18; 95% CI, 1.01--1.38; *P* = 0.04). The intercept of MR-Egger regression for these SNPs was not statistically significant (DBP: *P* = 0.461; SBP: *P* = 0.80), which means there is no evidence of directional pleiotropy. Therefore, DBP and SBP might act as mediators in the causal pathway from IS to AF and account for 6.7 and 7.4% of the total effect of IS on AF, respectively.

### The bidirectional causality between blood pressure and IS

Additionally, bidirectional MR analyses were performed to examine whether there existed the bidirectional causality between blood pressure and IS. We used 91 SNPs and 80 SNPs that were reported by the Neale Lab (Churchhouse & Neale, 2017) to be associated with DBP and SBP as the IVs, respectively, and found that increased DBP and SBP levels were associated with higher risk of IS (DBP: OR, 1.65; 95% CI, 1.27–2.14; *P* = 1.73 × 10^−4^. SBP: OR, 1.48; 95% CI, 1.17--1.87; *P* = 0.001). Therefore, the bidirectional causality between blood pressure and IS was verified. In other words, the causal association between blood pressure (DBP, SBP) and AF was simultaneously mediated by IS (Fig. 1b). Finally, we obtain that the mediated proportion of IS in the causal pathway from blood pressure (DBP, SBP) to AF were 13.9 and 14.1%, respectively.

### The bidirectional causality between AF and IS

We additionally performed MR of AF on IS to examine the bidirectional causality between AF and IS. The causal estimate using 103 SNPs (explaining 1.1% of exposure’s variance) as IVs showed that AF will increase the risk of developing IS (odds ratio [OR], 1.18; 95% confidence interval [CI], 1.08–1.28; *P* = 0.001). The intercept of MR-Egger regression for these 103 SNPs was not statistically significant (*P* = 0.737) so there is no evidence of directional pleiotropy. Thus, there are bidirectional causality between AF and IS and they are risk factors for each other. Details are briefly presented in Table 3 and the results of bidirectional MR analyses are listed in Additional file 2: Table S11-S12 and Additional file 1: Figure S7. There was > 80% power to detect all the causal associations and details were listed in Table 3.

**Table 3:** Causal estimates for the association between IS and AF and mediators

Exposure	Outcome	Causal estimate					
		Num of IVs	*R*^2^	Beta/OR	95% CI	PValue	Power
IS	AF	103	6.1%	1.05	1.02–1.07	1.3 × 10^−5^	0.84
IS	DBP	103	6.1%	0.02	0.001–0.034	2.9 × 10^−2^	–
DBP	AF	91	1.5%	1.18	1.03–1.35	1.2 × 10^−2^	1
IS	SBP	103	6.1%	0.02	0.006–0.034	3.1 × 10^−3^	–
SBP	AF	80	1.7%	1.18	1.01–1.38	4.0 × 10^−2^	1
DBP	IS	91	1.5%	1.65	1.27–2.14	1.7 × 10^−4^	1
SBP	IS	80	1.7%	1.48	1.17–1.87	9.3 × 10^−4^	0.99
AF	IS	103	1.1%	1.18	1.08–1.28	1.3 × 10^−4^	1

## Discussion

Although the underlying mechanisms between IS and AF have been discussed in previous studies (Rizos et al. 2015), causal association between IS and AF has never been reported. In this study, we used publicly available summary statistics from several genetic consortia to verify the causal association between IS and AF. We further explored pathway that might be involved in the association from IS to AF by a network MR analysis. We concluded that IS was associated with higher concentrations of SBP and DBP, which could further increase AF risk. In addition, we also found, the causal association from blood pressure to AF may be mediated by IS under similar Network Mendelian randomization framework above.

The risk of stroke in patients with AF is 3–5 times higher than in patients with non-AF (Wolf et al. 1978). AF has been consistently associated with IS in different cohorts (Sposato et al, 2015; Rizos et al, 2016; Manolio et al. 1996). Intuitively, uncoordinated myocyte activity can explain the impaired atrial contraction in AF, and according to Virchow’s triad, the resulting stasis of blood should increase the risk of thromboembolic. AF is a common cause of stroke, which is biologically plausible and is proven in our study. And what we are most interested in is the causal pathway from IS to AF. The brain exerts the greatest control over heart rhythm through the autonomic nervous pathway, which may be affected by cerebral lesions (i.e., IS) or systemic inflammation (Kamel et al, 2016). In patients with insular or other cortical acute ischemic stroke, sudden loss of autonomic or even central regulation can cause the arrhythmia stimulus in the intrinsic system, which in turn triggers focal discharges in the pulmonary veins and non-pulmonary veins. Ultimately paroxysmal atrial fibrillation occurred. Inflammatory mediators with elevated plasma concentrations during the acute phase of ischemic stroke may affect the intrinsic system, leading to the development of focal firing and subsequent AF in acute stroke patients. Other factors different from autonomic dysfunction and inflammation should be acknowledged as possibly implicated in the pathophysiology of poststroke AF (Sposato et al. 2014).

Our study demonstrates that blood pressure is a mediator from IS to AF. Biologically, blood pressure is related to the mechanism of AF’s occurrence. The autonomic nervous system and its sympathetic arm play important roles in the regulation of blood pressure. Their role in the short-term regulation of blood pressure, especially in responses to transient changes in arterial pressure, via baroreflex mechanisms is well known (Joyner et al. 2010). Besides, inflammation is associated with elevated blood pressure in the general population. A prospective cohort study shows that C-reactive protein (CRP) levels are associated with future development of hypertension, which suggests that hypertension is in part an inflammatory disorder (Sesso et al. 2004). However, how the autonomic nervous system and inflammation act on blood pressure to cause AF is unclear. Therefore, the role of autonomic nervous system, inflammation and blood pressure in the causal pathway from IS to AF and how their interaction in this pathway to cause AF needs further attention. Lattanzi et al. (2013) mentioned that raised blood pressure is common after acute stroke, whether of ischaemic or haemorrhagic type. Other studies suggest that elevated blood pressure is present during rehabilitation and training for stroke patients (Odden et al, 2015). In addition, Roetker et al. (2014) founded that plus pressure emerged as a significant independent risk factor for AF in a Multi-Ethnic Study of Atherosclerosis. Chen et al. (2014) indicated that central nervous system injuries often affect the autonomic nervous system, which plays an important role in the pathogenesis of AF. Necrotic cell death from stroke activates a systemic inflammatory response, which also plays a role in the origin of AF. Clinical observations support the hypothesis that stroke may trigger AF. Therefore, there are reasons believe that the causal association from IS to AF may be mediated by blood pressure.

Castillo et al. (2004) and Zhang et al. (2006) have reported positive and reverse association between elevated blood pressure on stroke severity, respectively. In this study, the bidirectional causality between blood pressure and IS was verified by directional MR analysis. Hypertension is the most important risk factor for all types of stroke, especially in China. Zhang X F et al. found that for each increase of 10 mmHg in systolic blood pressure, there was a 1.44-fold risk for IS in Chinese hypertensive patients. In addition, elevated blood pressure is present during rehabilitation and training for stroke patients. Our study provided a strong evidence that raised blood pressure in stroke patients increases the risk of atrial fibrillation and active acute blood pressure lowering can improve outcome in ischemic stroke patients (Carlberg et al. 1993).

We additionally performed MR for AF and IS subtypes (cardioembolic stroke (CE), large vessel disease (LVD), small vessel disease (SVD)) and results showed only a strong causal relationship from AF to cardiogenic stroke (Additional file 2: Table S12). Several large prospective epidemiological investigations suggested that other markers of left atrial dysfunction such as elevated N-terminal pro-Brain Natriuretic Peptide (NT pro-BNP) (Folsom et al. 2013), p-wave terminal force in lead V1 (PTFV1) of a 12-lead electrocardiogram (Lattanzi et al. 2017) and left atrial enlargement (Yaghi et al. 2015) are associated with cardioembolic stroke. Various studies have proved that NT-proBNP is increased in AF and proposed mechanisms are high frequency of atrial myocyte contraction and local atrial inflammation (Jayachandran and Johnson 2009). Goda T et al. found that PTFV1 on admission ECG is a strong, independent predictor of PAF in patients with acute ischemic stroke (Goda et al. 2017). Once AF occurs, LA dilation might progress due to either progressive heart disease, loss of the atrial systole, or both factors (Andersen et al. 1991). Therefore, the markers of left atrial dysfunction may play an important role in the causal mechanism between IS and AF. And further studies need to attention is the causal association among these markers, cardioembolic stroke and AF.

The main strength of our study is the large sample size accrued from the GWAS summary statistics, enabling us to examine the causal relationship among IS, risk factors, including DBP and SBP, and AF. In addition, the IVs that explained the largest variance of exposure without heterogeneity were selected by an iterative algorithm, which promises the effect of IS on traits is not a violation of the third assumption about pleiotropy. And we further valid the first assumption by the rule of thumb proposed by Staiger and Stock (1997) and test the third assumption by MR-Egger regression in the sensitive analysis. The random assortment of alleles at birth should rule out confounding factors in the association among IS, DBP and SBP, and AF. Another obvious major strength of using GWAS summary statistics with two sample MR is the increased statistical power, particularly when the outcome is a binary trait like AF. Besides, the bidirectional causality between blood pressure and IS was verified.

The limitations mainly concern the assumptions for two sample MR analyses. There is no sample overlap between the cohort of exposure ISGC (IS) and the outcome AFGen (AF), but there may still be individuals participating in multiple surveys that we cannot ascertain with available summary-level GWAS statistics. The test of InSIDE assumption in MR-Egger might be a problem. InSIDE assumption is that the effect of genetic variants on the exposure is independent of the direct effects of the genetic variants on the outcome, which is difficult to evaluate. Therefore, it is recommended to use individual data in one sample as much as possible to perform Mendelian randomization studies, so as to obtain higher accuracy and power. In addition, no matter in one-sample or two-sample MR analysis, potential violation of the second core assumption cannot be ruled out because of possible unmeasured confounders. While instrument strength can be verified by the calculation of F-statistics and pleiotropy can be tested by MR-Egger.

## Conclusion

In summary, we provided a causal diagram among blood pressure, IS and AF. We found that IS could increase the risk of AF and the effect of genetically determined IS on AF was partially mediated by blood pressure. Additionally, due to the bidirectional causality between blood pressure and IS, IS might also play a mediating role on the pathway between blood pressure and AF. Thus, raised blood pressure in stroke patients increases the risk of AF and active acute blood pressure lowering can improve outcome in IS patients, which is of significance in clinical application. Further validation of these findings is warranted based on large-scale longitudinal studies that repeat the measurements of IS, blood pressure, and AF.

## Supplementary information

**Additional file 1.** Methods details including Inverse-variance weighted method, MR-Egger method, Weak Instruments and Mediation analysis. Leave-one-out plots in the sensitive analysis.

**Additional file 2.** The basic characters of summary data used in this study. Details of instrumental invariables selected for each traits.

## Data Availability

The GWAS summary statistics datasets we used in this study were from the ISGC Consortium (https://strokegenetics.org/) for ischemic stroke; MAGIC Consortium (http://www.magicinvestigators.org/) for FastingInsulin; GIANT Consortium (https://www.broadinstitute.org/collaboration/giant/index) for body mass index, waist circumference and height; Neale Lab (http://www.nealelab.is/) for systolic and diastolic blood pressure; Astle W for white blood cell count; MRC-IEU Consortium (http://www.bristol.ac.uk/integrative-epidemiology/) for procalcitonin; AFGen Consortium (https://www.afgen.org/) for atrial fibrillation.

## Abbreviations

AF: Atrial fibrillation
AFGen: Atrial Fibrillation Consortium
BMI: Body mass index
DBP: Diastolic blood pressure
GIANT: Genetic investigation of anthropometric traits
GWAS: Genome-wide association study
IS: Ischemic stroke
ISGC: International Stroke Genetics Consortium
IV: Instrument variable
MAGIC: Meta-analyses of glucose and insulin-related traits consortium
MR: Mendelian randomization
MRC-IEU: MRC Integrative Epidemiology Unit
PCT: Procalcitonin
SBP: Systolic blood pressure
SNP: Single-nucleotide polymorphisms
WBC: White blood cell count
WC: Waist circumference
