# Supplementary material for: Exploring the causal pathway from ischemic stroke to atrial fibrillation: a network Mendelian randomization study
Source: Mol Med. 2020 Jan 15;26:7. doi: 10.1186/s10020-019-0133-y (PMC6964084; doi:10.1186/s10020-019-0133-y)
Supplement: Supplementary file 1 — Additional file 1. Methods details including Inverse-variance weighted method, MR-Egger method, Weak Instruments and Mediation analysis. Leave-one-out plots in the sensitive analysis. [file 10020_2019_133_MOESM1_ESM.pdf]

Additional file 1

Supplemental Methods Inverse-variance weighted method, MR-Egger method, Weak Instruments and Mediation analysis

Figure S1 Illustration of the MR analysis when a genetic variant is in linkage disequilibrium with a causal variant.
Figure S2 Illustration of the MR analysis when the population stratification exists.
Figure S3 Illustration of pleiotropic in the MR analysis.
Figure S4 Scatter plots showing the per-allele association with AF plotted against the per-allele association with IS before and after removing rs12646447.
Figure S5 Scatter plots showing the per-allele association with DBP plotted against the per-allele association with IS (a), SBP plotted against the per-allele association with IS (b), AF plotted against the per-allele association with DBP (c), AF plotted against the per-allele association with SBP (d), IS plotted against the per-allele association with DBP (e), IS plotted against the per-allele association with SBP(f), AF plotted against the per-allele association with IS(g).
Figure S6 Leave-one-out plots of MR estimate in causal effects of IS on AF before and after removing rs12646447.
Figure S7 Leave-one-out plots of MR estimate in causal effects of IS on DBP (a), IS on SBP (b), DBP on AF (c), SBP on AF (d), DBP on IS (e), SBP on IS (f) and AF on IS (g).

Supplemental Methods

Inverse-variance weighted method

Inverse-variance weighted (IVW) estimator can be calculated from summarized data. To some extent, IVW method is a fixed-effect meta-analysis, where the IV-specific causal estimates are the study-specific estimates, and the weights are the inverse-variance weights. The causal estimate from the IVW method (β_{IVW}) is calculated by the following equation:

$$\beta_{IVW} = \frac{\sum_{i=1}^{N} \beta_{IV-X}\beta_{IV-Y}\sigma_{IV-Y}^{-2}}{\sum_{i=1}^{N} \beta_{IV-X}\sigma_{IV-Y}^{-2}}$$

The standard error is estimated by:

$$se(\beta_{IVW}) = \frac{1}{\sum_{i=1}^{N} \beta_{IV-X}\sigma_{IV-Y}^{-2}}$$

where N is the total number of genetic variants, β_{IV-X} is the effects of i-th genetic variant on the exposure, β_{IV-Y} is the i-th genetic variant on the outcome, and σ_{IV-Y}^{-2} is the standard error of β_{IV-Y}.

MR-Egger method

MR-Egger method assume that the correlation between the genetic associations with the exposure (β_{IV-X}) and the direct effects of the genetic variants on the outcome (β_{IV-Y}) is zero, which refer as InSIDE (Instrument Strength Independent of Direct Effect). By fitting the linear model:

$$\beta_{IV-Y} \sim \alpha_{0E} + \alpha_E \beta_{IV-X}$$

where α_{0E} and α_E are the coefficients in the regression model. The slope and intercept represent the causal effect of exposure on outcome and the average pleiotropic effect across the genetic variants (the average direct effect of a variant with the outcome), respectively. The estimate coefficients from Egger regression is calculated from the following equation:

$$\alpha_E = \frac{\text{cov}(\beta_{IV-Y}, \beta_{IV-X})}{\text{var}(\beta_{IV-X})}, \quad \alpha_{0E} = \overline{\beta_{IV-Y}} - \alpha_E \overline{\beta_{IV-X}}$$

Weak Instruments

The F statistic is a measure of instrument strength. It is related to the proportion of variance in the exposure explained by the genetic variants (R^2), sample size (n) and the number of instruments (k) by the formula

$$F = \left(\frac{n\text{-}k\text{-}1}{k}\right)\left(\frac{R^2}{1-R^2}\right).$$

The commonly cited rule-of-thumb is that $F > 10$ avoids bias in IV analysis.

Mediation analysis

The association of IS with AF was mediated by DBP and SBP were tested in an additional analysis after DBP and SBP were identified as potential mediators. The total effect (risk ratio: RR) of 1 SD increase of IS on AF was 1.04 [log(RR)= 0.047]. The effect of 1 SD increase of IS on DBP was 0.019, and 1 unit increase in DBP was associated with AF was 1.18[log(OR) = 0.169]. Thus, the mediated effect of DBP was $0.019\times0.169=0.0032$. The mediated proportion was 0.0032/0.047 = 6.8%. In a similar way, we obtain the mediated proportion of DBP in the causal pathway of IS on AF was 7.4%.

In addition, the association of DBP with AF was mediated by IS and the association of SBP with AF was mediated by IS were tested in a post-hoc analysis. The mediated proportion of IS in the causal pathway of DBP on AF was 13.9%; the mediated proportion of IS in the causal pathway of SBP on AF was 14.1%. Details are listed in supplemental table 12.

Figure S1 Illustration of the MR analysis when a SNP is in linkage disequilibrium with a SNP.

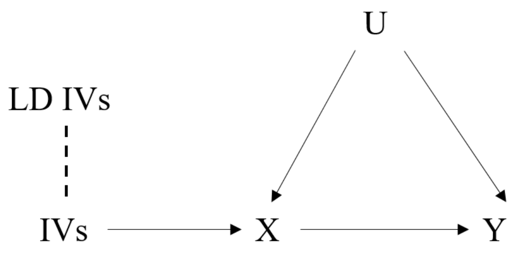

Linkage disequilibrium (LD) is the correlation between allelic states at different loci within the population. In Mendelian randomization studies, assumptions are violated if the genetic variant being used as an instrument is in linkage disequilibrium with a genetic variant that is related to the confounders or outcome.(X, modifiable exposure of interest; Y, outcome of interest; U, (unmeasured or measured with error), confounders; IV, instrumental variables)

Figure S2 Illustration of the MR analysis when the population stratification exists.

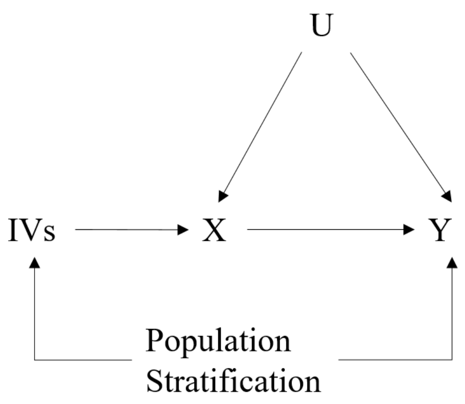

Population stratification occurs when there exist population subgroups that experience both different distributions of traits and have different frequencies of alleles of interest. This can result in a violation of assumption 3 and spurious associations between modifiable exposure and disease in the whole study population. (X, modifiable exposure of interest; Y, outcome of interest; U, (unmeasured or measured with error), confounders; IV, instrumental variables)

Figure S3 Illustration of pleiotropic in the MR analysis.

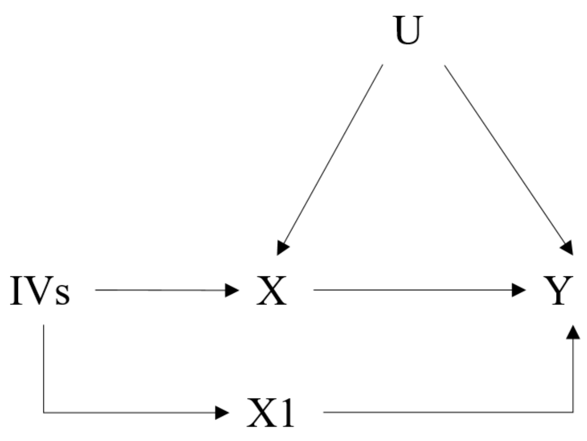

Pleiotropy refers to a genetic variant having multiple functions. The Mendelian randomization study will result in the violation of the assumptions 3 if the variant is associated with pleiotropic effects (X1) that do not (other than via the modifiable exposure of interest) influence the outcome; genetic variants with pleiotropic effects that do influence the outcome will, however, invalidate the Mendelian randomization approach.

Figure S4 Scatter plots showing the per-allele association with AF plotted against the per-allele association with IS before and after removing rs12646447.

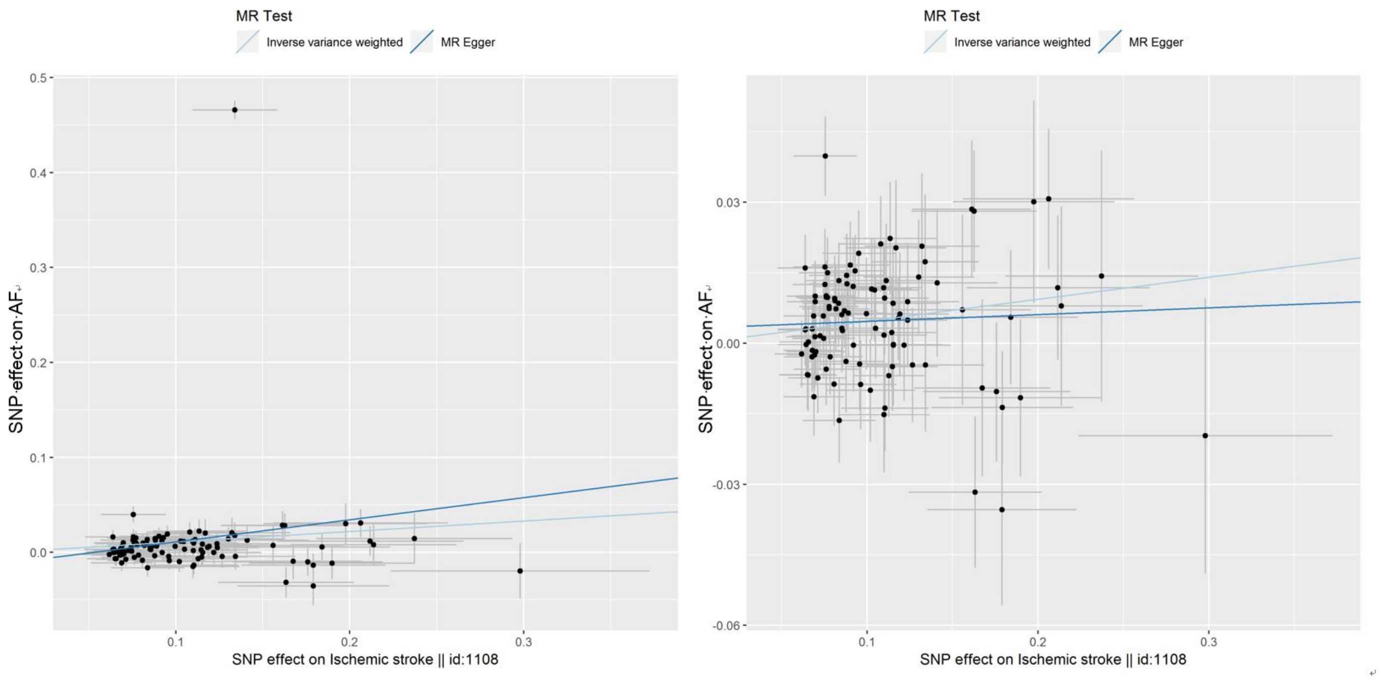

(a) Before removing rs12646447 (b) After removing rs12646447

Figure S5 Scatter plots showing the per-allele association with DBP plotted against the per-allele association with IS (a), SBP plotted against the per-allele association with IS (b), AF plotted against the per-allele association with DBP (c), AF plotted against the per-allele association with SBP (d), IS plotted against the per-allele association with DBP (e), IS plotted against the per-allele association with SBP(f).

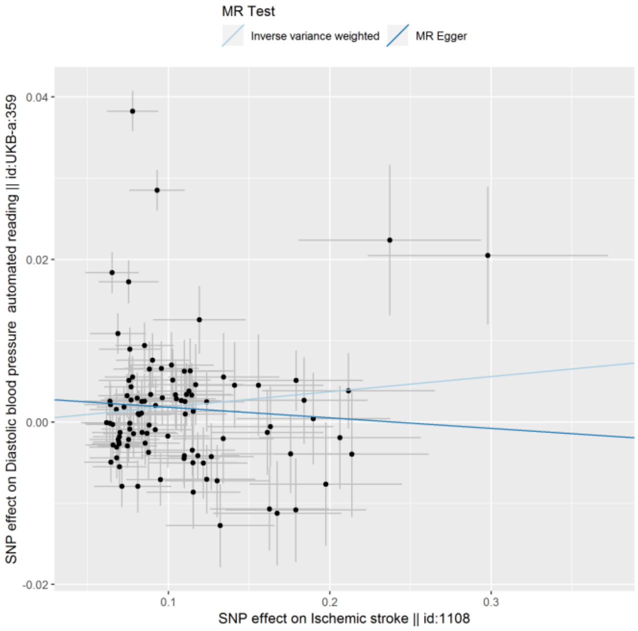

(a) IS on DBP

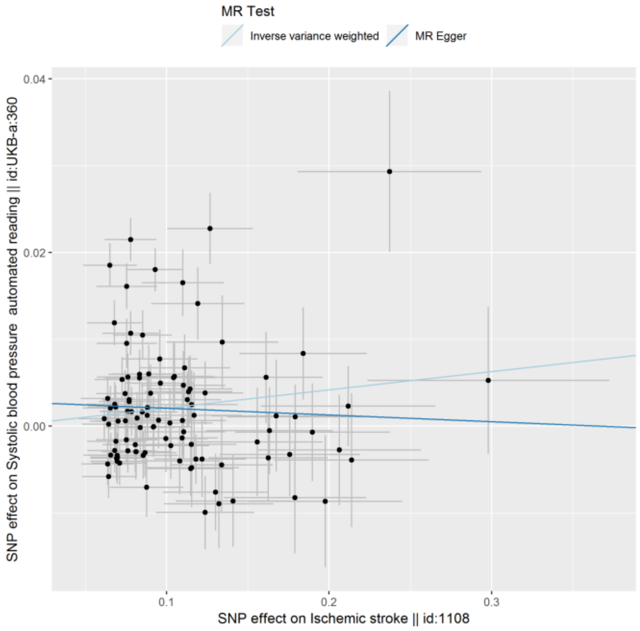

(b) IS on SBP

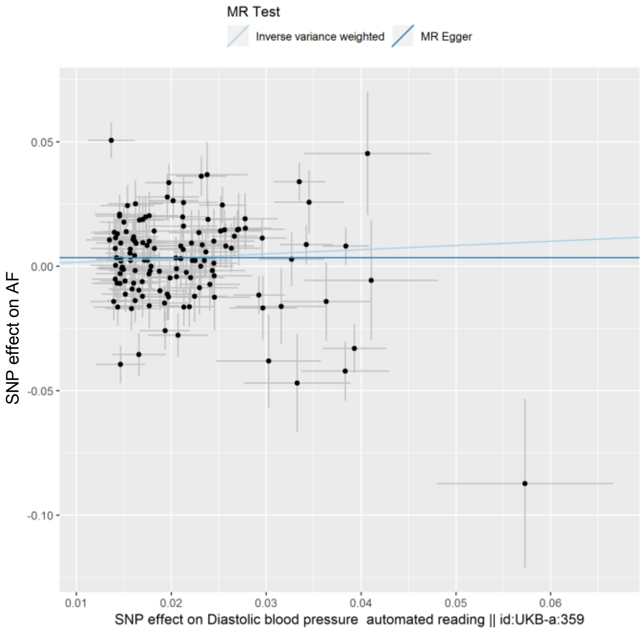

(c) DBP on AF

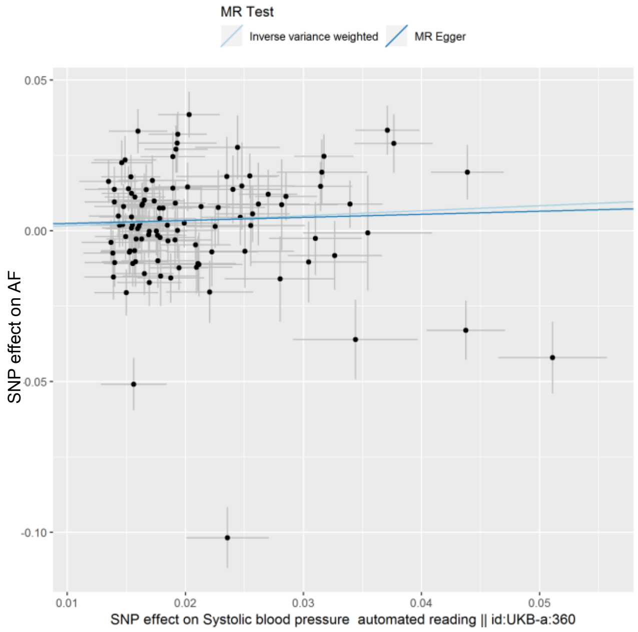

(d) SBP on AF

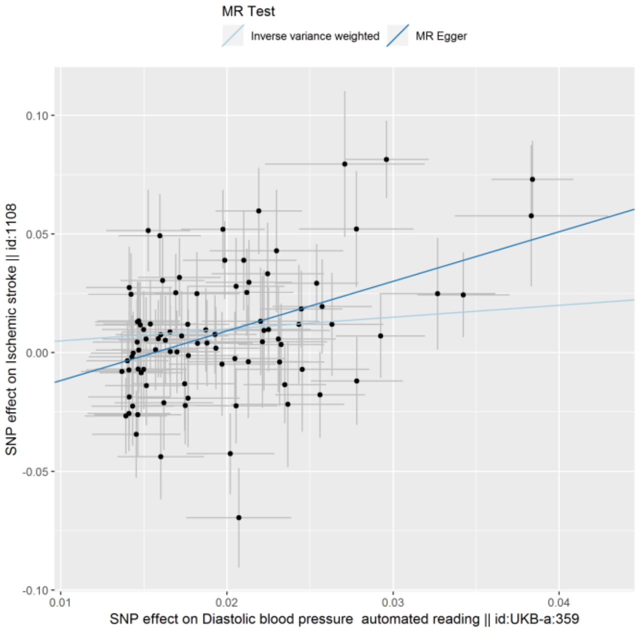

(e) DBP on IS

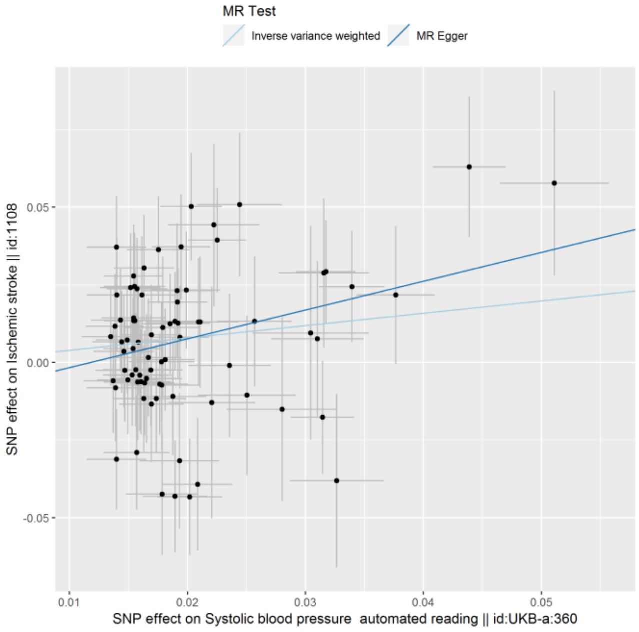

(f) SBP on IS

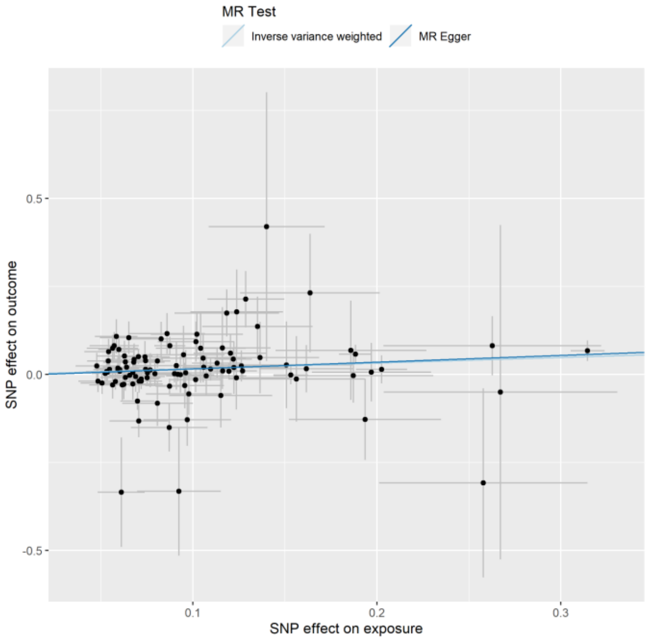

(g) AF on IS

Figure S6 Leave-one-out plots of MR estimate in causal effects of IS on AF before and after removing rs12646447.

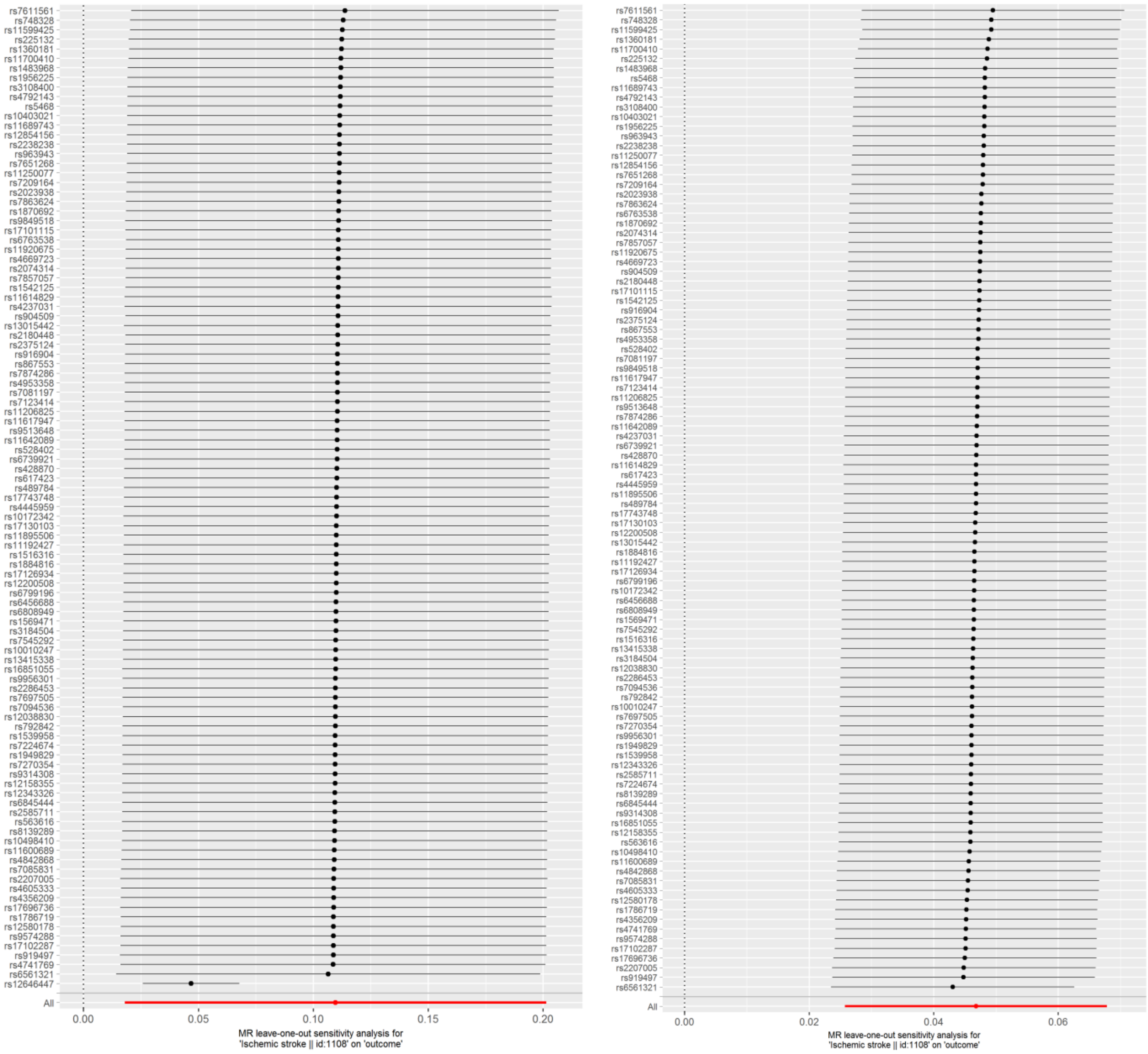

(a) Before removing rs12646447 (b) After removing rs12646447

Figure S7 Leave-one-out plots of MR estimate in causal effects of IS on DBP (a), IS on SBP (b), DBP on AF (c), SBP on AF (d), DBP on IS (e) and SBP on IS (f).

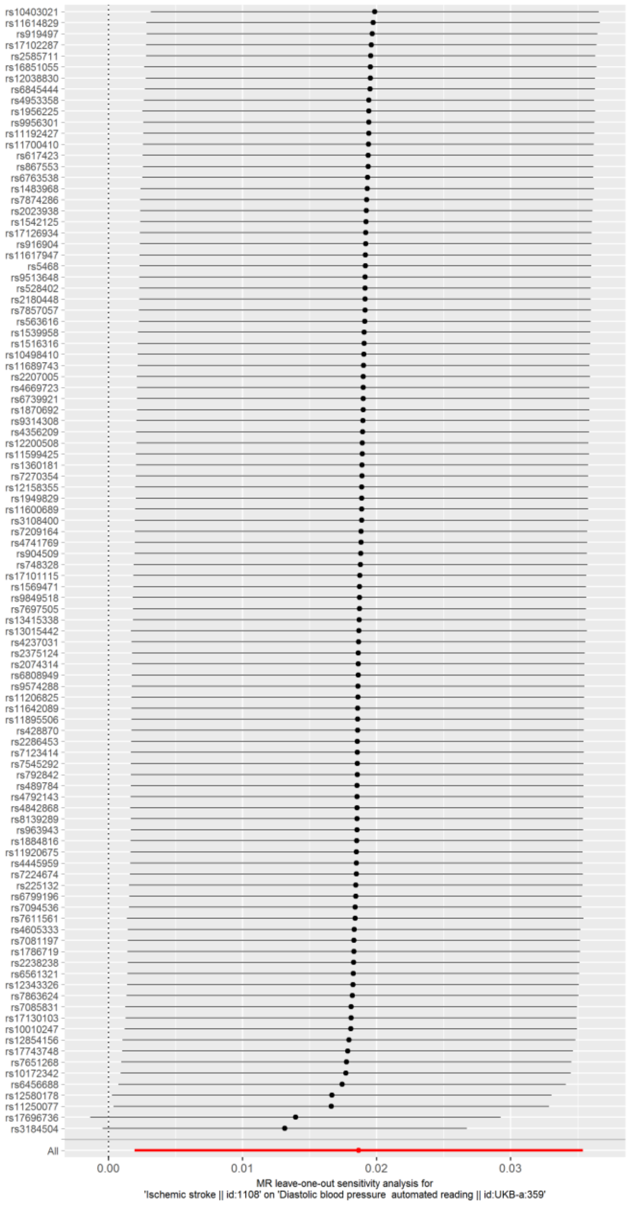

(a) IS on DBP

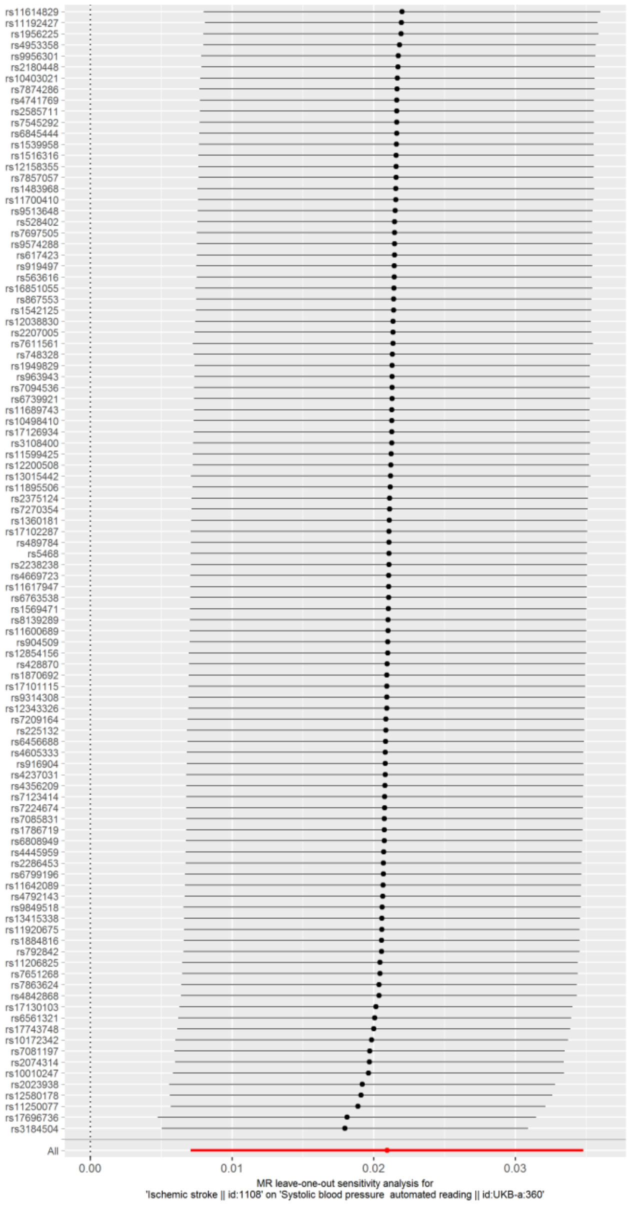

(b) IS on SBP

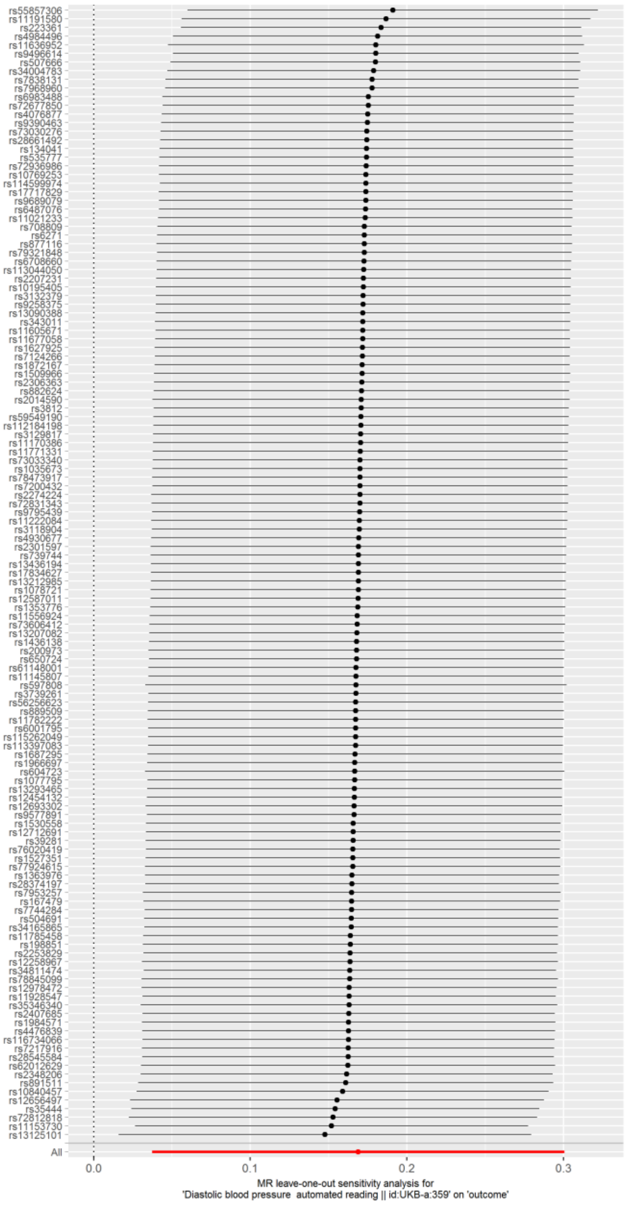

(c) DBP on AF

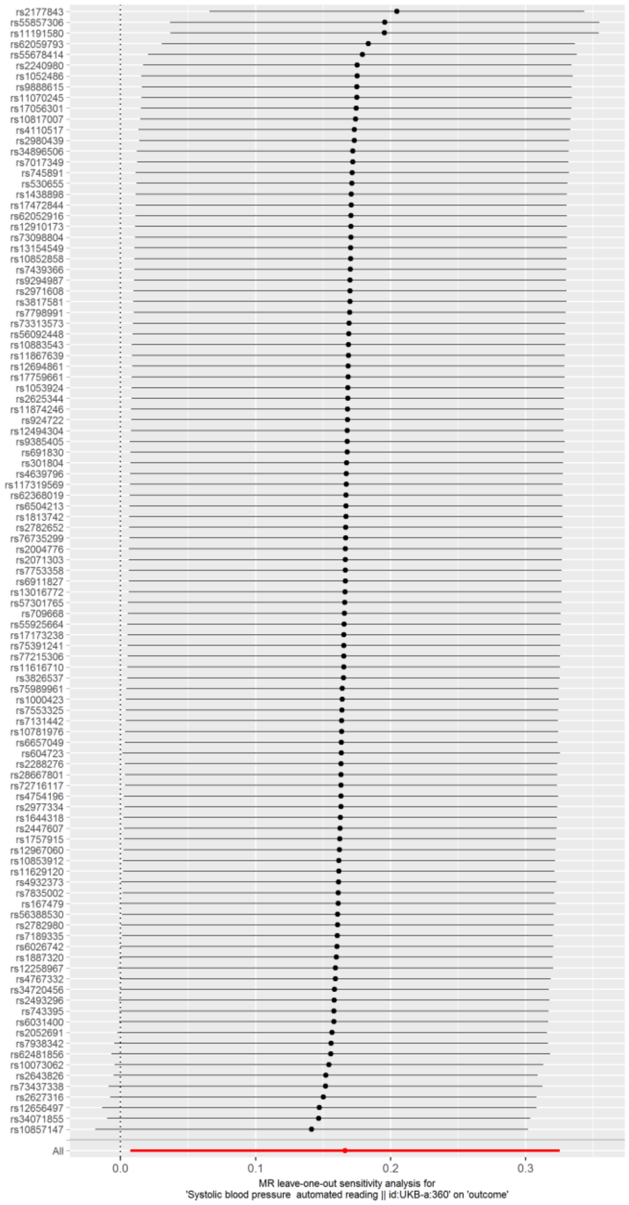

(d) SBP on AF

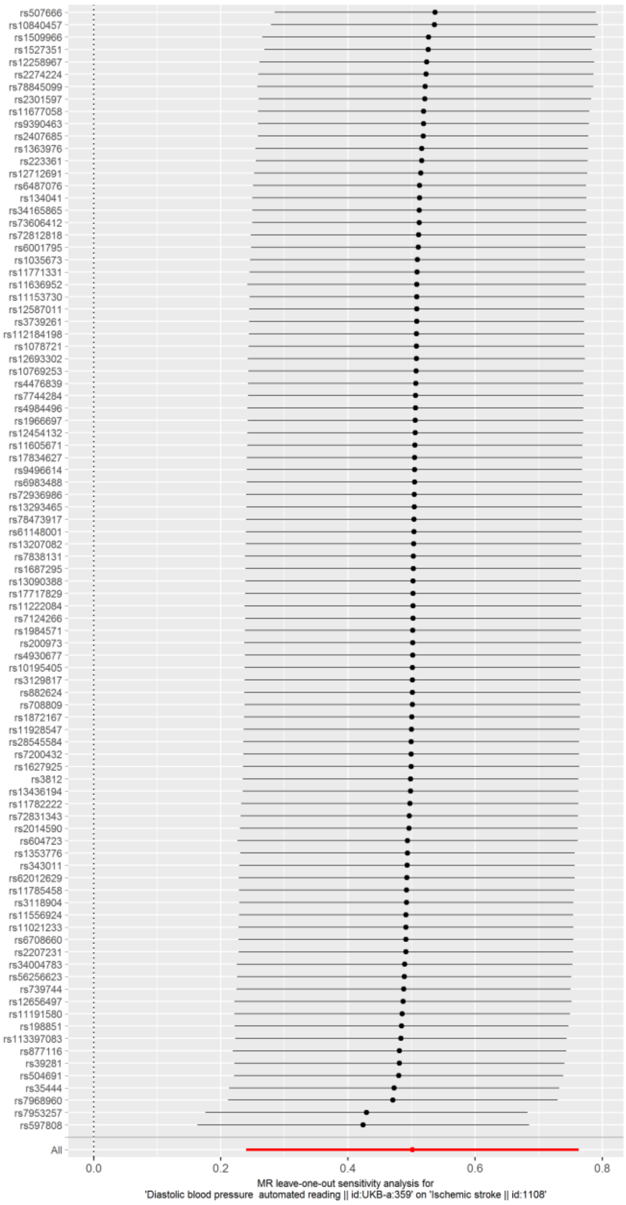

(e) DBP on IS

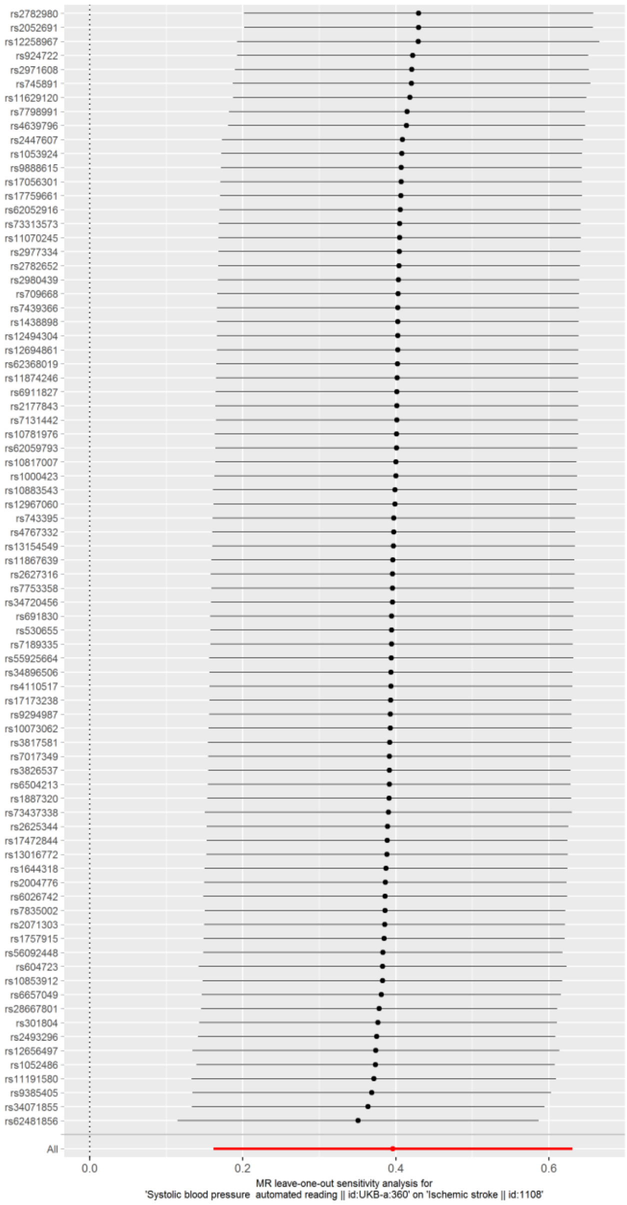

(f) SBP on IS

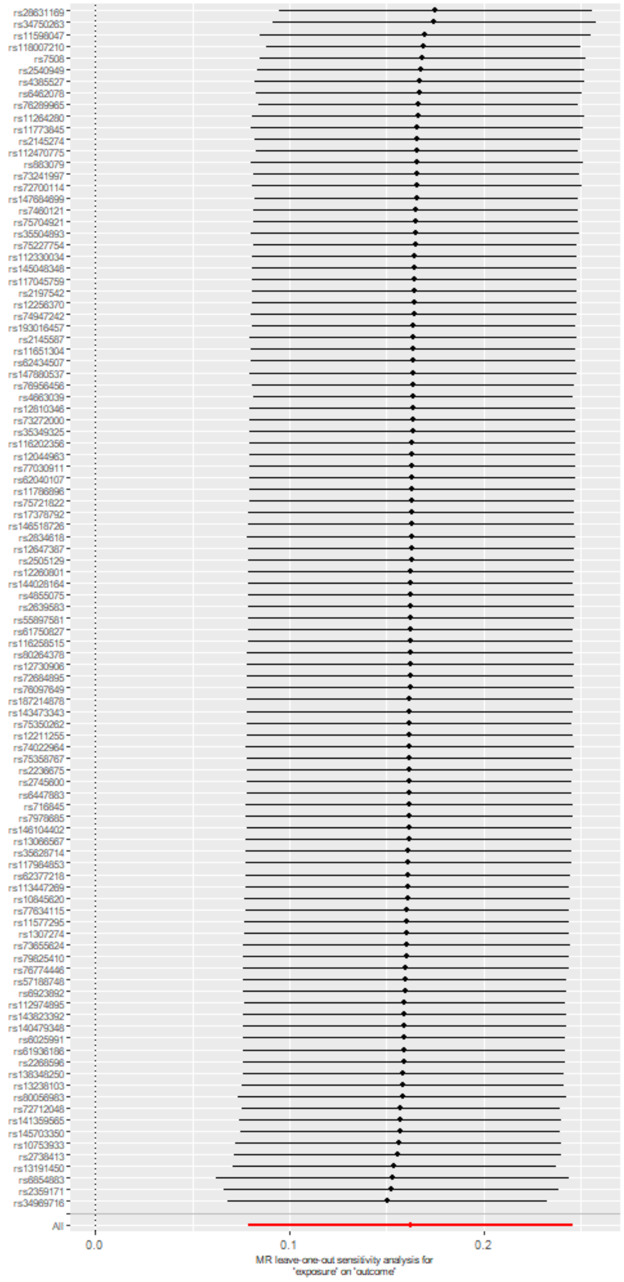
rs28631169
rs34750263
rs11598047
rs118007210
rs7508
rs2540949
rs4385527
rs6462078
rs76289965
rs11264280
rs11773845
rs2145274
rs112470775
rs883079
rs73241997
rs72700114
rs147684699
rs7460121
rs75704921
rs35504893
rs75227754
rs112330034
rs145048348
rs117045759
rs2197542
rs12256370
rs74947242
rs193016457
rs2145587
rs11651304
rs62434507
rs147880537
rs76956456
rs4863039
rs12810346
rs73272000
rs35349325
rs116202356
rs12044963
rs77030911
rs62040107
rs11786896
rs75721822
rs17378792
rs146518726
rs2834618
rs12647387
rs2505129
rs12260801
rs144028164
rs4855075
rs2639583
rs55897581
rs61750827
rs116258515
rs80264378
rs12730906
rs72684895
rs76097649
rs187214878
rs143473343
rs75350262
rs12211255
rs74022964
rs75358767
rs2236675
rs2745600
rs6447883
rs716845
rs7978685
rs146104402
rs13066567
rs35628714
rs117984853
rs62377218
rs113447289
rs10845620
rs77634115
rs11577295
rs1307274
rs73655624
rs79825410
rs76774446
rs57188748
rs6923892
rs112974895
rs143823392
rs140479348
rs6025991
rs61936186
rs2268596
rs138348250
rs13238103
rs80056983
rs72712048
rs141359565
rs145703350
rs10753933
rs2738413
rs13191450
rs6854883
rs2359171
rs34969716
All
0.0
0.1
0.2
MR leave-one-out sensitivity analysis for
'exposure' on 'outcome'

(g) AF on IS
